# Whole genome sequencing data of *Klebsiella pneumoniae* Ch1-39 isolated from chili powder

**DOI:** 10.1016/j.dib.2024.111050

**Published:** 2024-10-21

**Authors:** Mayra Paola Mena Navarro, Merle Ariadna Espinosa Bernal, Ana Laura Vega Rodríguez, Daniel Alejandro Ferrusca Bernal, Juan Enrique de Jesús López, Maria Carlota García Gutiérrez, Karla Isabel Lira De León, Bertha Isabel Carvajal Gaimez, David Gustavo García Gutiérrez, Alma Delia Bertadillo Jilote, Miguel Angel Ramos Lopez, José Luis Hernández Flores, Juan Campos Guillén

**Affiliations:** a Facultad de Química, Universidad Autónoma de Querétaro, Cerro de las Campanas S/N, Querétaro 76010, México; b Center for Advanced Biomedical Research, School of Medicine, Autonomous University of Queretaro, Campus Aeropuerto Carretera a Chichimequillas S/N, Ejido Bolaños, 76140 Santiago de Querétaro, Qro., México; c Facultad de Ciencias Naturales, Universidad Autónoma de Querétaro, Carretera a Chichimequillas S/N, Campus aeropuerto, Querétaro 76140, México; d Centro de Investigación y de Estudios Avanzados del IPN, Irapuato 36824, México

**Keywords:** *Klebsiella pneumoniae* Ch1-39, Complete genome, Antibiotic Resistance genes (ARGs), Virulence factor genes, Chili powder

## Abstract

*Klebsiella pneumoniae* Ch1-39 was isolated from chili powder elaborated at San Luis Potosí, México. This microorganism can be found in diverse ecological niches as water, soil, air, plants and hospital setting, it is considered as a relevant opportunistic pathogen causing several diseases and showing increasingly multi-resistance to antibiotics. The genome was sequenced on the Illumina NovaSeq platform and bioinformatic analyses were made at the Bacterial and Viral Bioinformatics Resource Center (BV-BRC). The genome consisted of 72 contigs with a total size of 5,410,125 bp, 5,361 protein coding sequences (CDS), a total of 6 rRNA and 76 tRNA with an average G + C content of 57.22 %. The genome data was deposited at National Center for Biotechnology Information (NCBI) under accession number Bioproject ID PRJNA1062060, Bio Sample ID SAMN40269967. The genome accession number was JBAWUH000000000.

Specifications Table

**Table:** 

Subject	Biological sciences
Specific subject area	Microbiology, Genomics, Bioinformatics
Data format	Raw, Filtered and analyzed
Type of data	Complete genome sequence in FASTA format Table(s), Figure(s)
Data collection	*Klebsiella pneumoniae* Ch1–39 was isolated using MacConkey agar selective medium from chili powder elaborated at San Luis Potosí, México. DNA was extracted using the ZymoBIOMICS^TM^DNA Miniprep Kit and sequenced using the Illumina NovaSeq platform. Bioinformatic analysis was carried out on BV-BRC platform for adapter trimming and quality filtering using Trim Galore and Fastq-Pair. Comprehensive genome analysis service at PATRIC for genome assembly, genome annotation and phylogenetic tree. The genome map was produced with platform Proksee. The AMR phenotype analysis showed resistance to ampicillin, clindamycin, dicloxacillin, erythromycin, penicillin, vancomycin and carbenicillin.
Data source location	Institution: Universidad Autónoma de Querétaro City/Town/Region: Querétaro, Qro. Country: México GPS coordinates: 20°35′28″N 100°24′36″O
Data accessibility	Analyzed data are provided in this report. assembly data is deposited on public repository. Repository name: *Klebsiella pneumoniae* Ch1-39 chromosome deposited in NCBI Data identification number: JBAWUH000000000 Direct URL to data: https://www.ncbi.nlm.nih.gov/nuccore/JBAWUH000000000 Database link: Bio Project: PRJNA1062060, Bio Sample: SAMN40269967

## 1 Value of the Data

• The complete genome sequence of *K. pneumoniae* Ch1-39 may be useful for comparative genomic studies with other *Klebsiella* species.
• This data is useful for the scientific community because it provides insights into the genome of an environmental bacterial isolate.
• The genome data can provide insights for the understanding of virulence and antibiotic genes with potential health risk to the chili powder consumers.
• This data is applicable for the monitoring of microbial food safety during the processing of the chili dried fruits into powder.

## 2 Background

Chili powder is produced from dried fruits of a wide selection of chili pepper plants (*Capsicum spp*.). It is an important condiment consumed around the world. However, during the processing of the dried fruits into powder, it can be contaminated by pathogenic microorganisms and can be a concern for public health risk during its consumption [1]. As such, the isolation of bacteria from chili powder is necessary to gain knowledge through of genome sequencing about virulence and antibiotic resistance genes. Until now, there is no available information of genomes of pathogenic microorganisms isolated from chili powder in Mexico. This work aims to contribute with a draft genome of *K. pneumoniae* Ch1-39 to provide genomic information to understand the potential health risk of this microorganism and avoid the spread in this important condiment.

## 3 Data Description

**Fig. 1:**
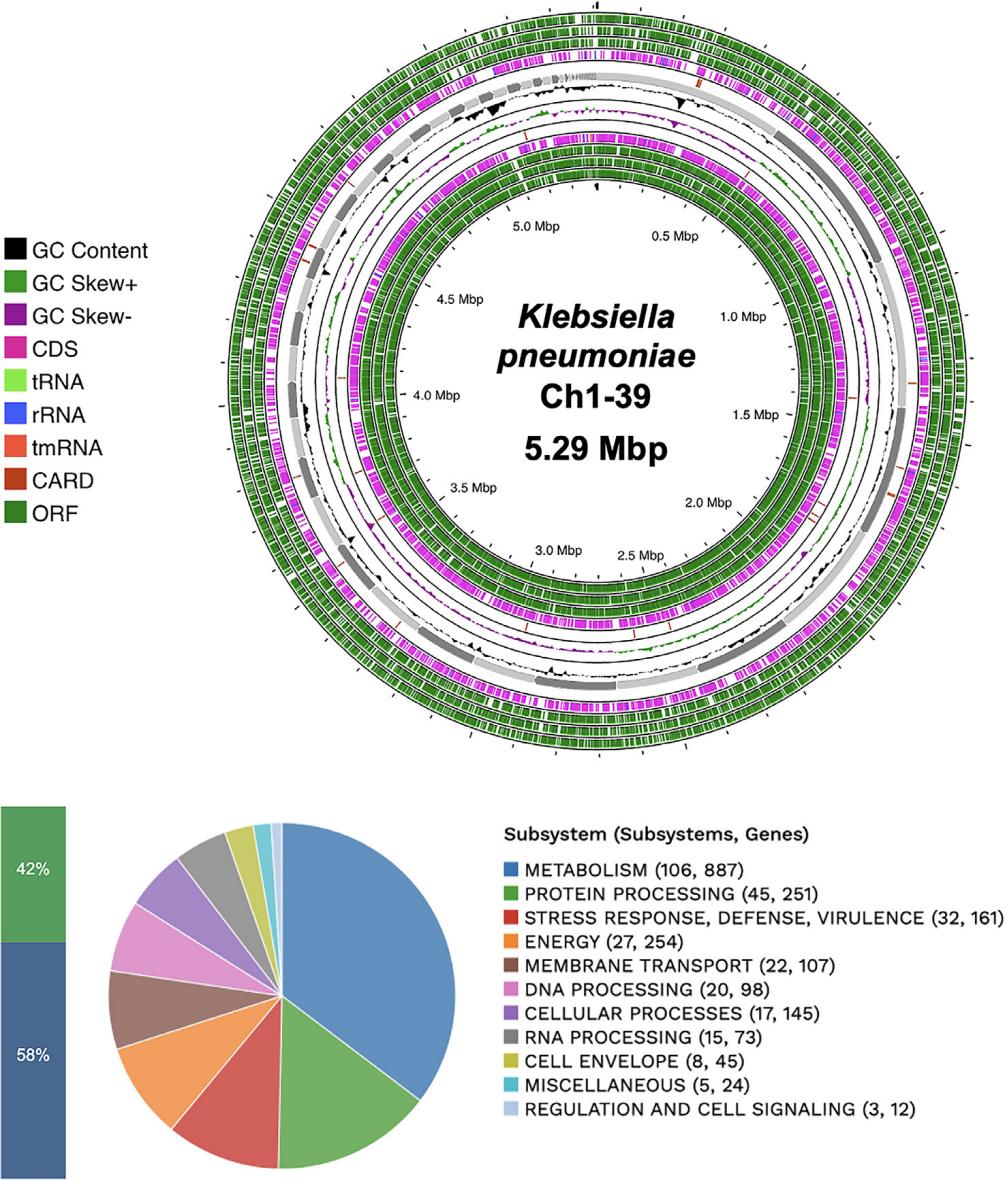
Circular genomic map and subsystems information for *K. pneumoniae* Ch1-39. From the outside to the center are the assembled contigs, ORF, CDS in the front strand, CDS on the reverse strand, RNA genes, CDS with similarity to known antibiotic resistance genes, CDS with similarity to virulence factors, GC content and GC skew. Distribution of the subsystems are displayed in the figure below. In subsystems coverage, 42 % indicates a total of 2619 genes and 58 % represent those not indicated in subsystem average with a total 2824 genes.

*K. pneumoniae* Ch1-39 was isolated from chili powder sample processed in the state of San Luis Potosí, central México. Table 1 summarizes genomic characteristics of *K. pneumoniae* Ch1-39 obtained in BV-BRC platform [2]. The analysis resulted in a genome length of 5410,125 bp. The genome sequence formed 72 contigs and an average G + C content of 57.22 %, with N50 length (the shortest sequence length at 50 % of the genome) of 347,819 bp. Using Rast tool kit (RASTtK) [3], the *K. pneumoniae* Ch1-39 was annotated using genetic code 11. The genome contains 5361 protein coding sequences (CDS), 76 transfer RNA (tRNA) genes and 6 ribosomal (rRNA) genes. The annotation describes other important features of the genome, including 727 hypothetical proteins and 4634 proteins with functional assignments. Virulence factors, antibiotic resistance genes, transporter genes and drug target genes are shown for each source. In addition, the distribution of the subsystems is described, which represents the main biological and metabolic processes (Fig. 1).

**Table 1:** Genomic description of *K. pneumoniae* Ch1-39.

Characteristics	Source	Total
Genome Length	PATRIC	5410,125 bp
Number of contigs	PATRIC	72
Number of proteins characterized	PATRIC	5361
Number of putative/hypothetical proteins	PATRIC	727
Number of rRNA genes	PATRIC	6
Number of tRNA genes	PATRIC	76
Number of proteins with pathway annotation	KEGG	1039
G + C %	PATRIC	57.22 %
N50 contig size (bp)	PATRIC	347,819 bp
Virulence factors	Victors	146
Virulence factors	VFDB	20
Antibiotic resistance genes	CARD	58
Antibiotic resistance genes	PATRIC	60
Antibiotic resistance genes	NDARO	4
Transporter genes	TCDB	585
Drug target genes	DrugBank	311

**Fig. 2:**
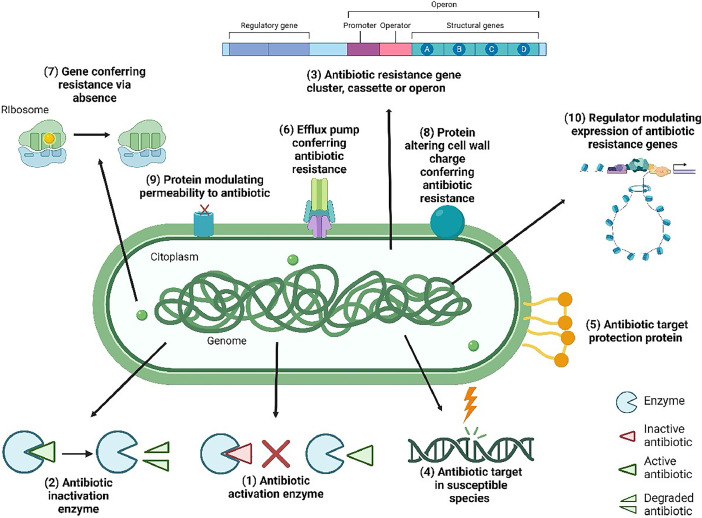
Genes predicting antibiotic resistance were grouped into ten mechanisms of action. (1) antibiotic activation enzyme, (2) antibiotic inactivation enzyme, (3) antibiotic resistance gene cluster, cassette or operon, (4) antibiotic target in susceptible species, (5) antibiotic target protection protein, (6) efflux pump conferring antibiotic resistance, (7) gene conferring resistance via absence, (8) protein altering cell wall charge conferring antibiotic resistance, (9) protein modulating permeability to antibiotic and (10) regulator modulating expression of antibiotic resistance genes. Created using Biorender.

Based on the genome analysis, genes predicting antibiotic resistance were grouped into ten mechanisms of action indicated by numbers in Fig. 2. The mechanism of action to (1) antibiotic activation enzyme is represented by the *katG* gene. The mechanism of action to (2) antibiotic inactivation enzyme is represented by SHV family. The mechanism of (3) antibiotic resistance gene cluster, cassette or operon is represented by *marA, marB, marR* genes. The mechanism of (4) antibiotic target in susceptible species is represented by *alr, ddl, dxr, eF-G, ef-Tu, folA, dfr, folP, gyrA, gyrB, inhA, fabl, Iso-tRNA, kasA, murA, rho, rpoB, rpoC, s10p, s12p* genes. The mechanism of (5) antibiotic target protection protein is represented by *bcrC* gene. The mechanism of (6) efflux pump conferring antibiotic resistance is represented by *acrAB-tolC, acrAD-tolC, acrEF-tolC, acrZ, emrAB-tolC, emrD, macA, macB, mdfA/cmr, mdtABC-tolC, mdtL, mdtM, sugE* and *tolC/opmH* genes. The mechanism of (7) gene conferring resistance via absence is represented by *gidB* gene. The mechanism of (8) protein altering cell wall charge conferring antibiotic resistance is represented by *gdpD* and *pgsA* genes. The mechanism of (9) protein modulating permeability to antibiotic is represented by *occD6/oprQ* and *oprB* genes. The mechanism of (10) regulator modulating expression of antibiotic resistance genes is represented by *acrAB-tolC, emrAB-tolC, H-NS* and *oxyR* genes.

Some of the genes that can be highlighted: are the SHV family that confer resistance to β-lactamases such as carbapenem, penam and cephalosporin. Resistance to the aminocoumarin antibiotic class is represented by the genes *gyrB* and *mdtABC-tolC*. The aminoglycoside resistance class is represented by the *acrAD-tolC* and *gidB* genes. The cephamycin, cephalosporin classes are represented by the *acrEF-tolC* gene. The diaminopyrimidine resistance class is represented by the *folA* and *dfr* genes. The fluoroquinolone class is represented by the genes *gyrA, emrAB-tolC* and *emrAB-tolC*. The macrolide class is represented by the *macA, macB* and *H-NS* genes. The peptide antibiotic class represented by the *bcrC, gdpD*, and *pgsA* genes. The class of resistance to tetracycline is represented by *marR, marA* and *acrAB-tolC* genes.

According to the reference database for bacterial virulence factors (VDFB), virulence-related genes are principally classified as adherence, invasion and virulence factor (Table 2).

**Table 2:** Potential virulence-related genes in *K. pneumoniae* Ch1–39 predicted by VFDB.

Gene	Product	Mechanism of action
*fimC*	Chaperone protein FimC	Adherence, Invasion
*ykgK/ecpR*	Transcriptional regulator EcpR	Adherence
*fimE*	Type 1 fimbriae regulatory protein FimE	Virulence Factor
*yagY/ecpB*	CFA/I fimbrial auxiliary subunit	
*yagX/ecpC*	CFA/I fimbrial subunit C usher protein	
*entS*	Enterobactin exporter EntS	
*kdsA*	2-Keto-3-deoxy-D-manno-octulosonate-8-phosphate synthase	
*fimH*	Protein FimH (regulates length and adhesion of type 1 fimbriae, and mediates mannose binding)	
*yagW/ecpD*	CFA/I fimbrial minor adhesin	
*yagV/ecpE*	CFA/I fimbrial chaperone	
*fepG*	Ferric enterobactin transport system permease protein FepG	
*fepD*	Ferric enterobactin transport system permease protein FepD	
*fimB*	Type 1 fimbriae regulatory protein FimB	
*fepC*	Ferric enterobactin transport ATP-binding protein FepC	
*fepB*	Ferric enterobactin-binding periplasmic protein FepB	
*fimA*	Type-1 fimbrial protein, A chain	
*yagZ/ecpA*	CFA/I fimbrial major subunit	
*entE*	2,3-dihydroxybenzoate-AMP ligase [enterobactin] siderophore @ 2,3-dihydroxybenzoate-AMP ligase of siderophore biosynthesis	
*entB*	Isochorismatase [enterobactin] siderophore / Apo-aryl carrier domain of EntB @ Isochorismatase of siderophore biosynthesis	
*entA*	2,3-dihydro-2,3-dihydroxybenzoate dehydrogenase [enterobactin] siderophore @ 2,3-dihydro-2,3-dihydroxybenzoate dehydrogenase of siderophore biosynthesis	

**Fig. 3:**
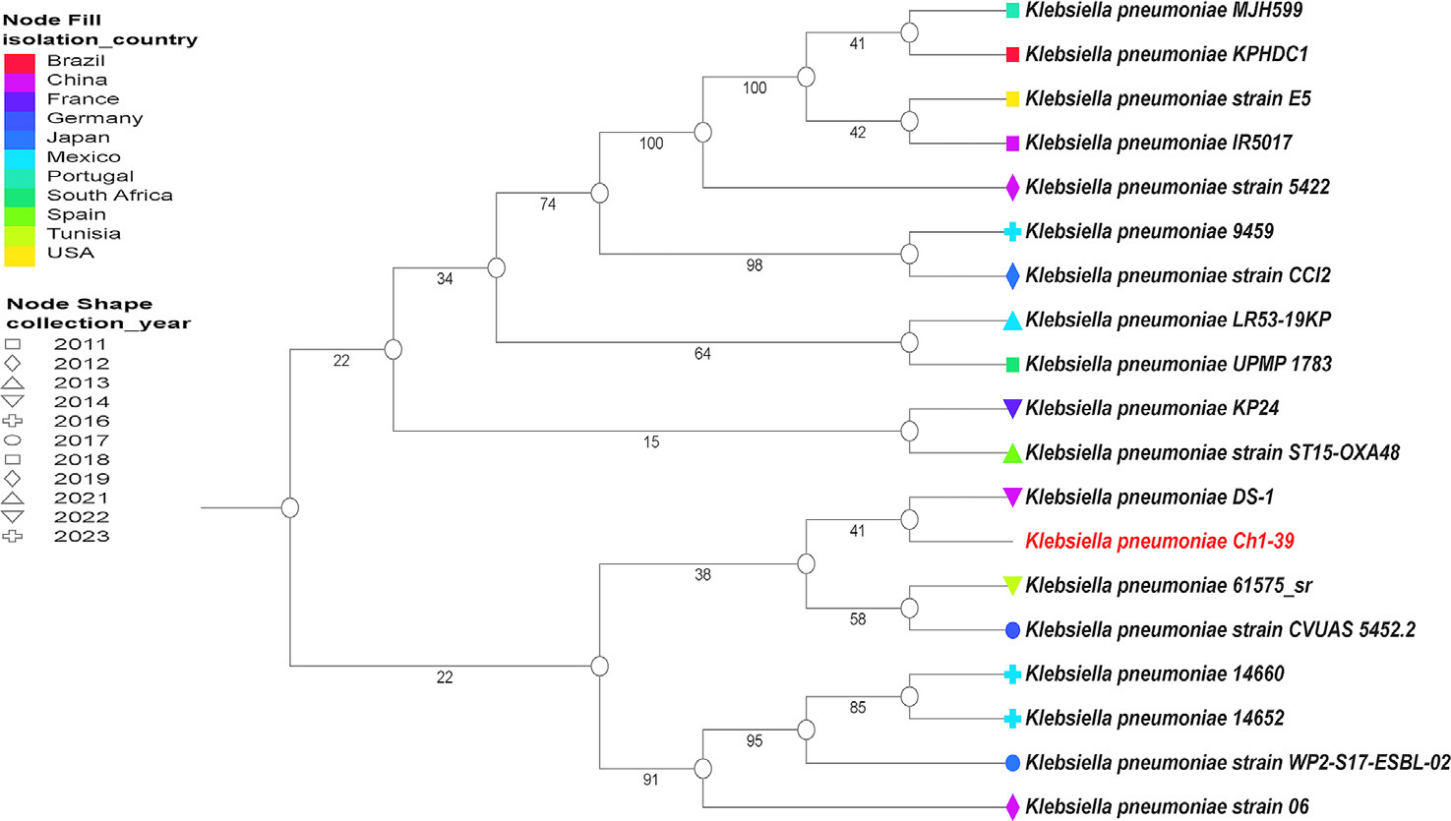
Phylogenetic tree including clinical and environmental strains of *K. pneumoniae* Ch1-39. The Codon Tree pipeline of BV-BRC was used to generate bacterial phylogenetic trees with *Klebsiella* genus strains. The following strains were included: *K. pneumoniae* MJH599 (573.64640), *K. pneumoniae* KPHDC1 (573.57106), *K. pneumoniae* IR5017 (573.56497), *K. pneumoniae* strain E5 (573.14977), *K. pneumoniae* strain 5422 (573.1345), *K. pneumoniae* 9459 (573.56896), *K. pneumoniae* strain CCI2 (573.34158), *K. pneumoniae* LR53-19KP (573.51887), *K. pneumoniae* 61,575_sr (573.64625), *K. pneumoniae* KP24 (573.54327), *K. pneumoniae* strain ST15-OXA48 (573.29101), *K. pneumoniae* UPMP 1783 (573.43879), *K. pneumoniae* DS-1 (573.53759), *K. pneumoniae* WP2-S17-ESBL-02 (573.32411), *K. pneumoniae* strain CVUAS 5452.2 (573.39775), *K. pneumoniae* 14,652 (573.63146), *K. pneumoniae* 14,660 (573.56900), *K. pneumoniae* strain 06 (573.32125). The color and figure of the node represent country and year collection for each insolated strain.

The phylogenetic tree of *K. pneumoniae* Ch1-39 is shown in Fig. 3. The data showed that Ch1-39 is closely related to *K. pneumoniae* DS-1 that was isolated of sewage from a treatment plant in China (BioSample: SAMN32958782), *K. pneumoniae* 61,575_sr which was isolated from sewage of wild pigeons in Tunisia (BioSample: SAMN37505229) and *K. pneumoniae* CVUAS 5452.2 which was isolated from calf in Germany (BioSample: SAMN12766886).

The AMR phenotype analysis showed resistance to ampicillin (10 µg), clindamycin (30 µg), dicloxacillin (1 µg), erythromycin (15 µg), penicillin (6 µg), vancomycin (30 µg) and carbenicillin (100 µg), while it showed sensitivity to cephalothin (30 µg), cefotaxime (30 µg), ciprofloxacin (5 µg), gentamicin (10 µg), tetracycline (30 µg), sulfamethoxazole/trimethoprim (25 µg), amikacin (30 µg), chloramphenicol (30 µg), netilmicin (30 µg), nitrofurantoin (300 µg), norfloxacin (10 µg), cefazolin (2 µg), levofloxacin (2 µg), meropenem (1 µg), cefepime (4 µg), ceftazidime (1 µg), ceftriaxone (1 µg), tigecycline (2 µg), cefuroxime (4 µg), ertapenem (0.5 µg), imipenem(1 µg) and tobramycin (4 µg) (Table 3).

**Table 3:** Antibiotic susceptibility of *K pneumoniae* Ch1-39.

Antibiotic	Phenotype	Antibiotic	Phenotype	Antibiotic	Phenotype
Ampicillin	R	Ciprofloxacin	S	Erythromycin	R
Cephalothin	I	Clindamycin	R	Gentamicin	S
Cefotaxime	S	Dicloxacillin	R	Penicillin	R
Tetracycline	S	Amikacin	I	Netilmicin	I
Sulfamethoxazole/Trimethoprim	I	Carbenicillin	R	Nitrofurantoin	I
Vancomycin	R	Chloramphenicol	I	Norfloxacin	I
Amoxicillin/Clavulanic Acid	S	Cefepime	S	Cefuroxime	S
Ampicilin/Sulbactam	S	Ceftazidime	S	Ertapenem	S
Cefazolin	S	Ceftriaxone	S	Imipenem	S
Levofloxacin	S	Piperacillin-Tazobactam	S	Tobramycin	S
Meropenem	S	Tigecycline	S		

S=Sensitive; I=Intermediate; R=Resistant

The biochemical analysis results are summarized in Table 4.

**Table 4:** Biochemical properties of *Klebsiella pneumoniae* Ch1-39.

Biochemical Test	Result	Biochemical Test	Result
Glucose	Positive	Urea	Positive
Sucrose	Positive	Lysine	Positive
Sorbitol	Positive	Esculin	Positive
Rhamnose	Positive	Voges-Proskauer	Positive
Inositol	Positive	Citrate	Positive
Adenosine	Positive	Maltose	Positive
Melibiose	Positive	Raffinose	Positive
TSI (H_2_S)	Negative	Indole	Negative
Arginine	Negative	Tartrate	Negative
Ornithine	Negative	Acetamide	Negative

## 4 Experimental Design, Materials and Methods

### 4.1 Sample collection and microbial isolation

With the objective of isolating pathogenic bacteria from chili powder, samples from different geographical regions of México were assayed. From one chili powder sample elaborated at San Luis Potosí, bacterial colonies were obtained using MacConkey agar selective medium and incubated for 24 h at 37 °C. Based on phenotype, colonies were subcultured and grown on a tryptic soy agar (TSA) medium (Difco Laboratories, Detroit, MI, USA) supplemented with 100 µg/mL of ampicillin and incubated at 37 °C for 24 h to obtain colonies with beta-lactamase activity. From these colonies, strain Ch1-39 was selected for characterization.

### 4.2 Identification and characterization by MicroScan panel

All biochemical tests were performed according to standard procedures [4]. According to the manufacturer's instructions, a concentration of 1×10⁶ CFU/mL of bacterial suspension was carried out using the Prompt™ inoculation system (Beckman Coulter, Brea, CA, USA). Then, the bacterial suspension was transferred to the panels using a MicroScan Renok (Beckman Coulter, Brea, CA, USA), which delivers 100 µL of bacterial suspension to each well of the panel (Neg Combo Panel Type 68). Mineral oil was added to the wells containing glucose (GLU), urea (URE), lysine (LYS), hydrogen sulfide (H₂S), arginine (ARG), Ornithine (ORN), and decarboxylase base (DCB). The panels were incubated for 24 h at 35 °C under aerobic conditions, with the panels covered to prevent evaporation. Reagents for biochemical revelation were added, and the panels were read using an automated system MicroScan AutoSCAN-4 (Beckman Coulter, Model AS4). The LabPro software calculated the genus and specie, as well as microbial susceptibility, by generating a biochemical profile code, which was used to provide identification with probability scores of ≥85 %.

### 4.3 Genome sequencing, assembly and annotation

Genomic DNA of the *K. pneumoniae* Ch1-39 strain was extracted using a ZymoBIOMICS^TM^ DNA Miniprep Kit (Zymo Research, Irvine, CA, USA). Genomic DNA sample was profiled with shotgun genomic sequencing. Sequencing libraries were prepared with an Illumina® DNA Library Prep Kit (Illumina, San Diego, CA, USA) and the final library was sequenced on the platform NovaSeq® (Illumina, San Diego, CA, USA) at Zymo Research, Irvine, CA, USA. All bioinformatic analyses were made using the pipelines at the Bacterial and Viral Bioinformatics Resource Center (BV-BRC); the Fastq Utilities Service, the comprehensive genome analysis service at PATRIC and the RAST tool kit (RASTtk) for genome annotation [[2], [3], [4], [5], [6], [7]]. The Codon Tree pipeline of BV-BRC was used to generate the bacterial phylogenetic tree. It uses reference genomes identified with Mash/MinHash. Then, proteins and gene sequences were selected from PATRIC global protein families (PGFams) and aligned with MUSCLE v5. Concatenated alignment was analyzed with RaxML v8.2.11 (Randomized Axelerated Maximum Likelihood) with 100 rounds of fast bootstrapping. These sequences data including clinical and environmental strains of *Klebsiella pneumoniae* specifically strains E5 (573.14977), IR5017 (573.56497), MJH599 (573.64640), KPHDC1 (573.57106), strain 5422 (573.1345), 9459 (573.56896), strain CCI2 (573.34158), LR53-19KP (573.51887), DS-1 (573.53759), 61,575_sr (573.64625), strain WP2-S17-ESBL-02 (573.32411), KP24 (573.54327), 14,660 (573.56900), 14,652 (573.63146), strain 06 (573.32125), strain ST15-OXA48 (573.29101), strain CVUAS 5452.2 (573.39775), UPMP 1783 (573.43879) and Ch1–39 (573.65430). The circular genome map was constructed using the Proksee platform using Proksee Assemble, CGView Builder, CARD RGI and Prokka [8,9]. DNA sequences were deposited in NCBI as BioProject ID PRJNA1062060. The genome accession number was JBAWUH000000000.

### 4.4 Antibiotic sensitivity test

The antibiotic sensitivity test was carried out using the disk diffusion technique and microdilution test using a MicroScan based on the CLSI guide (Clinical & Laboratory Standards Institute: CLSI Guidelines) [10]. Antibiotic discs (Oxoid) containing clindamycin (30 µg), dicloxacillin (1 µg), erythromycin (15 µg), penicillin (6 µg), tetracycline (30 µg), vancomycin (30 µg), amikacin (30 µg), ampicillin (10 µg), carbenicillin (100 µg), cephalothin (30 µg), cefotaxime (30 µg), ciprofloxacin (5 µg), chloramphenicol (30 µg), gentamicin (10 µg), netilmicin (30 µg), nitrofurantoin (300 µg), norfloxacin (10 µg), sulfamethoxazole/trimethoprim (25 µg), cefazolin (2 µg), levofloxacin (2 µg), meropenem (1 µg), cefepime (4 µg), ceftazidime (1 µg), ceftriaxone (1 µg), tigecycline (2 µg), cefuroxime (4 µg), ertapenem (0.5 µg), imipenem(1 µg) and tobramycin (4 µg) were used. The culture plates were incubated at 37 °C for 18–24 h and the measurements were recorded.

## Limitations

Not applicable

## Ethics Statement

This work does not involve human subjects or animal subjects. The authors declare that this manuscript is original work and has not been published elsewhere.

## CRediT Author Statement

**Mayra Paola Mena Navarro:** Conceptualization, Methodology, Software; **Merle Ariadna Espinosa Bernal:** Data curation, writing- original draft; **Ana Laura Vega Rodríguez:** Writing- original draft, Methodology; **Daniel Alejandro Ferrusca Bernal:** Methodology, Formal analysis; **Juan Enrique de Jesús López:** Visualization, Investigation; **Maria Carlota García Gutiérrez:** Writing- original draft, Methodology; **Karla Isabel Lira De León:** Writing- original draft, Resources, Methodology; **Bertha Isabel Carvajal Gamez:** Writing- original draft, Methodology; **David Gustavo García Gutiérrez:** Writing-review & editing; **Alma Delia Bertadillo Jilote:** Writing-review & editing; **Miguel Angel Ramos Lopez:** Writing-review & editing; **José Luis Hernández Flores:** Resources, Writing-review & editing; **Juan Campos Guillen:** Validation, Supervision, Resources, Writing-review & editing, Supervision.

## Data Availability

NCBIKlebsiella pneumoniae strain CH1-39, whole genome shotgun sequencing project (Original data).

## Appendix Supplementary materials

Image, application 1
